# 

**Published:** 1876-08

**Authors:** 


AUGUST, 1876.

### THE AMERICAN JOURNAL OF DENTAL SCIENCE.

PUBLISHED MONTHLY.

$2.50 PER ANNUM.

EDITED BY

F. J. S. GORGAS, M. D., D. D. S.

VOL. 10.—THIRD SERIES.—No. 4.

BALTIMORE:
SNOWDEN & COWMAN.
LONDON:
TRUBNER & CO., 60 PATERNOSTER ROW.
1876.

PAYABLE IN ADVANCE.

